# The role of MALAT1 correlates with HPV in cervical cancer

**DOI:** 10.3892/ol.2014.1996

**Published:** 2014-03-24

**Authors:** YAN JIANG, YUEHUI LI, SHUJUAN FANG, BINYUAN JIANG, CHANGFEI QIN, PINGLI XIE, GUOHUA ZHOU, GUANCHENG LI

**Affiliations:** Cancer Research Institution, Xiangya Medical School, Central South University, Changsha, Hunan 410078, P.R. China

**Keywords:** metastasis-associated lung adenocarcinoma transcript 1, cervical cancer, human papilloma virus, cell proliferation

## Abstract

Cervical cancer, the second most common type of cancer in women worldwide, is responsible for >275,100 mortalities each year and is associated with high-risk human papilloma virus (HR-HPV). HPVs have two important oncogenes, E6 and E7, which have crucial roles in malignant transformation in cervical cancer. Metastasis-associated lung adenocarcinoma transcript 1 (MALAT1) is a long non-coding RNA originally identified in non-small cell lung cancer. Previous studies have revealed that MALAT1 is expressed in numerous tissue types, and is significant in maintaining the normal function of the body. However, it also appeared to be notably upregulated in numerous carcinoma types compared with adjacent non-cancerous tissues. In the present study, it was identified that MALAT1 expression was upregulated in cervical cancer cell lines compared with normal cervical squamous cell samples. Further study into the effect of MALAT1 on cellular phenotype revealed that MALAT1 was able to promote cell migration and proliferation. Of note, it was revealed that the expression of MALAT1 was decreased with the knockdown of HPV16 E6/E7 in CaSki cells. Furthermore, the investigations in clinical samples also revealed that MALAT1 was expressed in HPV-positive cervical squamous cells, but not in HPV-negative normal cervical squamous cells. These results indicate that HPV correlates with MALAT1 deregulation in cervical cancer.

## Introduction

Cervical cancer is the second leading cause of cancer-associated mortality in women worldwide, responsible for >275,100 deaths each year, with the mortality rate on the rise in a number of developing countries (1). Cervical squamous cell carcinoma (cervical SCC) is one of the most frequent types of cervical cancer, accounting for 80–90% of all cases. A large number of studies have confirmed that persistent infection of high-risk human papillomavirus (HR-HPV) is a critical and indispensible risk factor for cervical SCC and the development of precancerous lesions (2). The high-risk HPVs contain two important oncogenes, E6 and E7, which contribute to oncogenesis of cervical SCC by silencing the tumor-suppressive p53 and Rb proteins, eventually resulting in cell cycle disorder and malignant transformation (3–5). Although the underlying pathogenesis of cervical SCC has been identified, the molecular mechanisms in its progression have not yet been fully elucidated.

Long non-coding RNAs (lncRNAs) are non-coding transcripts that are >200 nucleotides in length that have recently emerged as important molecules in both normal development and tumorigenesis (6,7). Studies have demonstrated that these lncRNAs have an important role in numerous biological processes, including X-chromosome inactivation, genomic imprinting, chromatin modification, gene transcription and splicing (8–11). A large group of lncRNAs have exhibited deregulated expression in human cancer types and appeared to have specific functional roles in tumor progression. HOTAIR is a 2.2-kb lncRNA, and its expression level was identified to be associated with breast cancer metastases and mortality (12). Panzitt *et al* identified that HULC contributed to early hepatocellular tumorigenesis and may act as a promising biomarker in cancer diagnostics (13). Furthermore, lincRNA-p21 and PANDA were reported to regulate cell apoptosis in carcinomas through a p53 gene regulatory pathway (14,15).

Metastasis-associated lung adenocarcinoma transcript 1 (MALAT1), also known as nuclear-enriched abundant transcript 2, is a highly evolutionary conserved lncRNA with a full length of 8708 nt (16). MALAT1 is a highly abundant nucleus-retained RNA that localizes to nuclear speckles, a sub-nuclear domain enriched in pre-mRNA splicing factors and affects alternative splicing of pre-mRNAs through modulating the cellular distribution and activity of serine arginine dipeptide-containing SR splicing factors (17–19). Several recent studies have demonstrated that lncRNA MALAT1 is upregulated in several solid tumor types and contributes to tumor cell proliferation, apoptosis, migration and invasion. The first study that linked MALAT1 to cancer was in non-small cell lung cancer (NSCLC) patients in 2003. The authors identified that MALAT1 was a prognostic parameter for the survival of stage I lung carcinoma patients, and that its expression was higher in NSCLC with metastasis than that without (16). Later studies in hepatocellular carcinoma (HCC) revealed that MALAT1 was upregulated compared with normal liver tissue, and that the depletion of MALAT1 in HepG2 cells reduced cell viability, motility and invasiveness. Clinical analysis further proved that MALAT1 was an independent prognostic factor for HCC recurrence following liver transplantation (20,21). A large number of studies have certified the specific functions of MALAT1 in other solid tumors, but the molecular mechanism underlying its effects remains unclear (22–26).

## Materials and methods

### Cervical cancer cell lines and specimens

Human cervical cancer cell lines, HeLa (HPV18-positive), CaSki (HPV16-positive), SiHa (HPV16-positive) and HCC94 (HPV16-positive), as well as immortal human keratinocyte HaCaT cells, were grown in RPMI-1640 medium (Gibco-BRL, Carlsbad, CA, USA) supplemented with 10% fetal bovine serum (HyClone, Logan, UT, USA). All cells were cultured in a humidified atmosphere containing 5% CO_2_ at 37°C. A total of 40 cases of HPV-positive normal and abnormal cervical squamous epithelium specimens and 24 cases of HPV-negative normal cervical squamous epithelium were collected from patients who had undergone cervical Thinprep cytological test and high risk human papillomavirus (HR-HPV) detection at Xiangya Hospital of Central South University (Changsha, China). The samples were processed and stored in RNAlater RNA stabilization reagent (Qiagen, Hilden, Germany) at −20°C until RNA extraction. This study was approved by the ethics committee of the Cancer Research Institute, Central South University, (Changsha, China). Written informed consent and approval were obtained from each patient.

### RNA isolation from cells and specimens

Isolation of cell total RNA was performed using TRIzol reagent (Invitrogen Life Technologies, Carlsbad, CA, USA) according to manufacturer’s instructions. The total RNA samples were isolated using Total RNA kit I (Omega Bio-Tek, Inc., Norcross, GA, USA) according to manufacturer’s instructions. All of the RNA samples were examined for integrity and purity by an ultraviolet spectrophotometer (OD260/OD280) (SmartSpec 3000 UV Vis Spectrophotometer, Bio-Rad, Hercules, CA, USA).

### Reverse transcription-polymerase chain reaction (RT-PCR) analysis

A total of 1 μg RNA was absorbed for semi-quantified RT using an RevertAid First Strand cDNA Synthesis kit (Thermo Fisher Scientific, Inc., Pittsburgh, PA, USA) according to the manufacturer’s instructions. All primers for PCR were designed by Primer 5.0 (Premier, Palo Alto, CA, USA) and detected in NCBI Blast. Primers for MALAT1 detection: forward, AGCGGAAGAACGAATGTAAC and reverse, GAACAGAAGGAAGAGCCAAG; primers for CDK4: forward, GGAGTGTTGGCTGTATCTTTGC and reverse, CGGATTACCTTCATCCTTATGT; primers for CDK6: forward, TGGTCGTCACGCTGTGGTACAG and reverse, GCAGGTGGGAATCCAGGTTTTC; primers for cyclinD1: forward, GCATCTACACCGACAACTCC and reverse, CTCCTCCTCCTCTTCCTCCT; primers for cyclinE: forward, GCTTATTGGGATTTCATCTTTA and reverse, TCTGTGGGTCTGTATGTTGTGT; primers for HPV16 E6: forward, CCACCCAGAAAGTTACCACA and reverse, TGCAACAAGACATACATCGA; and primers for HPV16 E7: forward, TGGAGATACACCTACATTGCAT and reverse, CCATTAACAGGTCTTCCAACGT. The PCR conditions were one cycle of 94°C for 5 min, followed by 27 cycles of 94°C for 30 sec, 58°C for 30 sec and 72°C for 60 sec with an extension of 68°C for 10 min. The PCR products were visualized on 1% agarose gels stained with ethidium bromide and quantified by ImageJ software (NIH, Bethseda, MD, USA).

### Construction of expression vectors of MALAT1 and transfection

Total RNA was extracted from HeLa cells and the amplification of two fragments in MALAT1 (NR_002819.2) was performed using primer M1 (4481–5481): forward, GTTGTTTGGATATGGTAGTGTGTGG and reverse, ATA AGCACTTATCCCTAACATGCAA [introduced with XhoI site (forward) and an BamHI site (reverse), respectively]; and primer M2 (6419–7260): forward, GAGTGCTTGGCTCTTCCTTCTG and reverse, ACCTGTTTTCCTCATTTTGTCC [introduced with XhoI site (forward) and an Bsp120I site (reverse), respectively]. The PCR products of the MALAT1 gene were purified by a Gel Extraction kit (Omega Bio-Tek, Inc.) following 1% agarose gel electrophoresis, then double digested and ligated into the eukaryotic expression vector pEGFP-C1. The recombinant plasmids, pEGFP-C1/M1 and pEGFP-C1/M2, were transformed into competent *Escherichia coli* DH5α and then positive clones were identified by PCR, double digestion and DNA sequencing. For transfection, the recombinant plasmids were transfected into cells using Lipofectamine 2000 transfection reagent (Invitrogen Life Technologies) according to manufacturer’s instructions. Then, for transient transfection, the cells were harvested following 48 h and, for stable transfection, G418 was added as a screening reagent and a limiting dilution method was used to select the monoclones. The positive clone was detected by RT-PCR.

### Cell proliferation assay

Cell proliferation assay was determined using an Cell Counting kit-8 (CCK-8; Beyotime Institute of Biotechnology, Haimen, China) based on WST-8 [2-(2-methoxy-4-nitrophenyl)-3-(4-nitrophenyl)-5-(2,4-disulfophenyl)-2H-tetrazolium]. Cells were seeded in 96-well plates at a density of 10^3^ cells/well with serum-free medium in a total volume of 100 μl, and then washed with phosphate-buffered saline (PBS). The medium was replaced with DMEM (Gibco-BRL) supplemented with 1% fetal bovine serum (FBS; Hyclone) the next day. Then, WST-8 (10 μl) was added to each well and incubated for 2 h at 37°C, every 24 h for 7 days. The optical density (OD) was measured at 450 nm in an SM-3 automatic enzyme-linked immune analyzer (TianShi, Beijing, China).

### Cell cycle analysis

Cells were trypsinized, washed twice in PBS, counted and then collected following fixation in 70% ethanol overnight at 4°C. A total of 1×10^6^ cells were suspended and stained with 500 μl propidium iodide solution (Beyotime Institute of Biotechnology) for 30 min in the dark at 37°C. FACScan flow cytometry instrument (Becton-Dickinson, Franklin Lakes, NJ, USA) was used to analyze cell cycles. The CellQuest program (Becton-Dickinson) was used for data analysis.

### Wound healing assay

Cells were cultured in 6-well plates. A wound was scratched with a 200-μl pipette tip and captured when the monolayer cells reached subconfluency. The cells were washed three times with PBS and cultured in DMEM medium supplemented with 1% FBS and captured at different time points (24 or 48 h). The relative migration rate (%) of the cells was measured and quantified by the distance of cell migration divided by the distance measured at 0 h.

### Statistical analysis

Experimental data are presented as the mean ± standard deviation for three or more individual experiments. All statistical analyses were performed using a two-tailed Student’s t-test or one-way analysis of variance (SPSS 17.0; SPSS, Inc., Chicago, IL, USA). P<0.05 was considered to indicate a statistically significant difference. The diagrams were drawn by GraphPad Prism 5 (GraphPad Software, San Diego, CA, USA).

## Results

### MALAT1 expression in human cervical cancer cell lines

**Figure 1:**
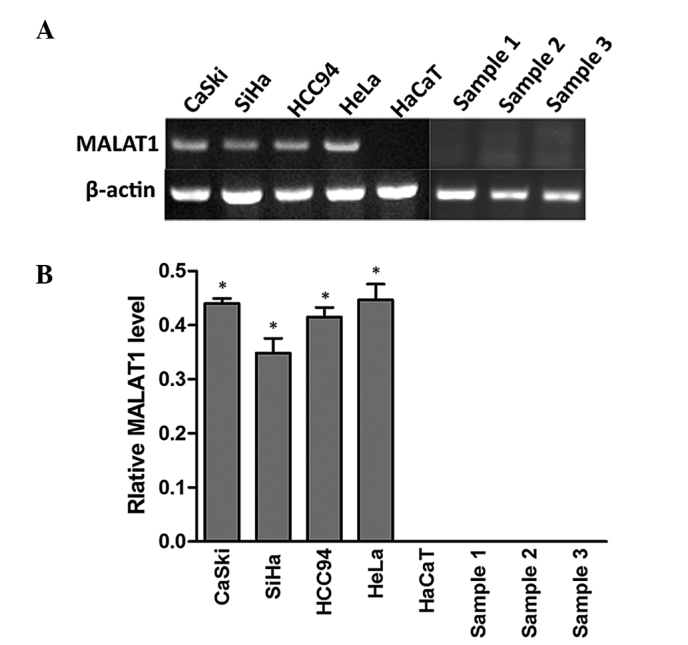
MALAT1 expression in human cervical cancer cell lines. (A) The expression of MALAT1 in HeLa, CaSki, SiHa, HCC94, HaCaT and normal cervical squamous cell samples was measured by reverse transcription-polymerase chain reaction. (B) Bar graph of the relative expression of MALAT1. β-actin was used as a loading control. ^*^P<0.05 compared with sample 1. MALAT1, metastasis-associated lung adenocarcinoma transcript 1.

To examine MALAT1 expression levels in cervical cancer, RT-PCR was used to detect the expression of MALAT1 in cervical cancer cell lines (HeLa, CaSki, SiHa and HCC94), immortal human keratinocyte HaCaT cells and in three cases of normal cervical squamous cells. As demonstrated in Fig. 1, MALAT1 was expressed in all cervical cancer cell lines but not in HaCaT cells and normal samples. This suggested that MALAT1 was activated in cervical cancer and may have an important role in tumorigenesis.

### Construction of stable MALAT1-overexpressing and -underexpressing cells

**Figure 2:**
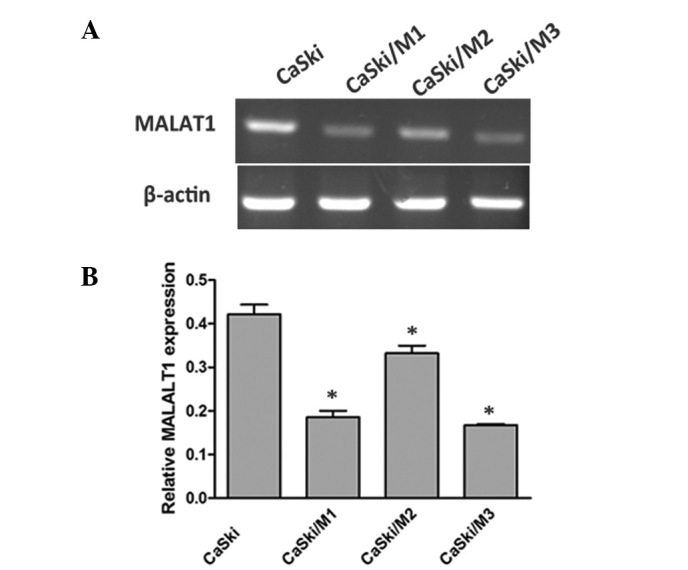
Downregulation of MALAT1 in CaSki cells. (A) Reverse transcription-polymerase chain reaction analysis of MALAT1 expression in stable cell lines CaSki/si-M compared with CaSki/C (vector-control) and CaSki (blank group). (B) Bar graph of the relative expression of MALAT1. β-actin was used as a loading control. ^*^P<0.05 compared with CaSki. MALAT1, metastasis-associated lung adenocarcinoma transcript 1.

**Figure 3:**
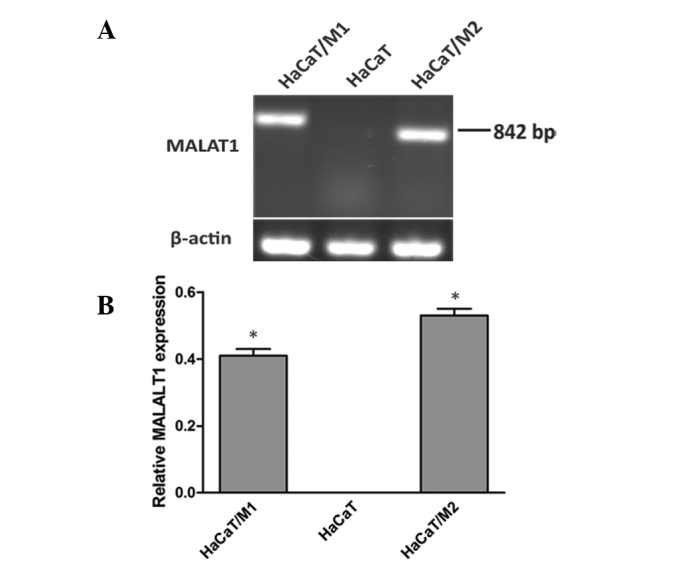
Upregulation of MALAT1 in HaCaT cells. (A) Reverse transcription-polymerase chain reaction analysis of MALAT1 expression in stable cell lines HaCaT/M1 and HaCaT/M2 compared with HaCaT (blank group). (B) Bar graph of the relative expression of MALAT1. ^*^P<0.05, compared with CaSki and HaCaT, respectively. β-actin was used as a loading control. MALAT1, metastasis-associated lung adenocarcinoma transcript 1.

To investigate the functional role of MALAT1, gain-of-function studies were conducted using HaCaT cells transfected with two fragments of MALAT, respectively (HaCaT/M1, HaCaT/M2) and loss-of-function in CaSki cells transfected with MALAT1 shRNA expression vector pRNAT-U6.1/Neo encoding a small hairpin RNA directed target sequences of MALAT1 5′-GACCTTGAAATCCATGACG-3′. The underexpression and overexpression efficiency are demonstrated in Figs. 2 and 3, respectively, and CaSki/M3 was selected as the MALAT1 downregulation group (CaSki/si-M) for the following experiments.

### MALAT1 affects cell migration and proliferation capability

**Figure 4:**
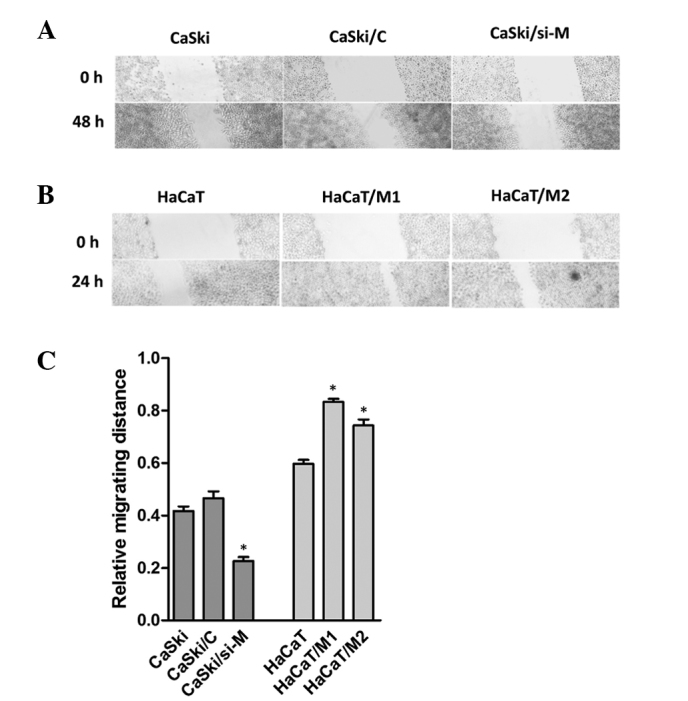
Effect of MALAT1 expression on cell migration. (A and B) CaSki, CaSki/C, CaSki/si-M and HaCaT, HaCaT/M1, HaCaT/M2 cells were performed with wound healing assay, the migrated distance was measured following 48 or 24 h. (C) Relative measurement of migration distance. ^*^P<0.05, compared with CaSki and HaCaT, respectively. MALAT1, metastasis-associated lung adenocarcinoma transcript 1.

To investigate whether MALAT1 impacted cell migration, a wound healing assay was performed. As demonstrated in Fig. 4, the migratory speed of CaSki/si-M was markedly slower than that of control cells, following 48 h, and the migration distance was also shorter in HaCaT cells than in the HaCaT/M1 or HaCaT/M2 cells following 24 h. These data suggested that MALAT1 may promote cell migration.

**Figure 5:**
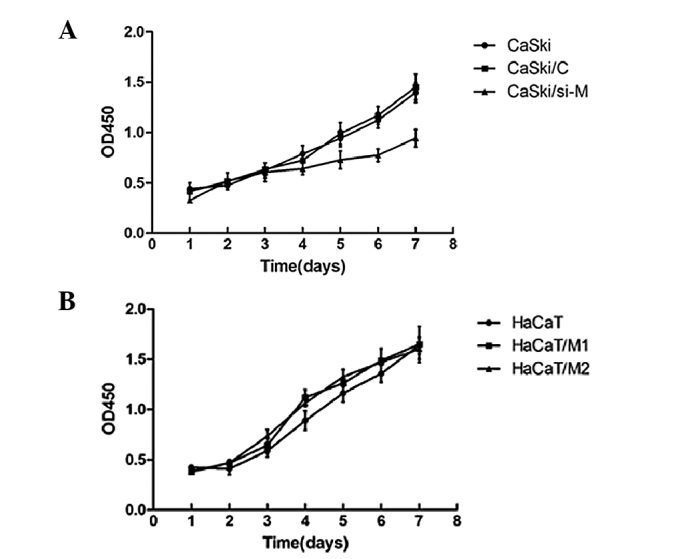
Effect of MALAT1 expression on cell proliferation. (A) Proliferation of CaSki, CaSki/C and CaSki/si-M cells was determined by CCK-8 every 24 h for 7 days. (B) Proliferation of HaCaT, HaCaT/M1 and HaCaT/M2 cells was determined by CCK-8 every 24 h for 7 days. MALAT1, metastasis-associated lung adenocarcinoma transcript 1; CCK-8, Cell Counting kit-8.

The CCK-8 assay revealed that cell proliferation was significantly inhibited in CaSki/si-M in comparison with non-transfectants (CaSki) and vector-control transfectants (CaSki/C) in CaSki cells (Fig. 5A). However, no significant difference was detected among the HaCaT/M1, HaCaT/M2 and HaCaT cells (Fig. 5B). These data indicate that downregulation of MALAT1 decreased cell proliferation ability in CaSki cells.

**Figure 6:**
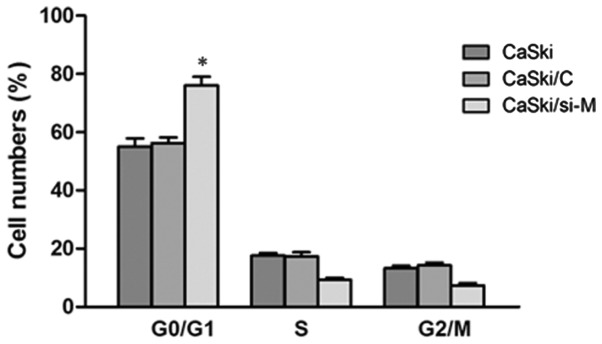
Analysis of cell cycle in CaSki, CaSki/C and CaSki/si-M cells. ^*^P<0.05, compared with CaSki.

**Figure 7:**
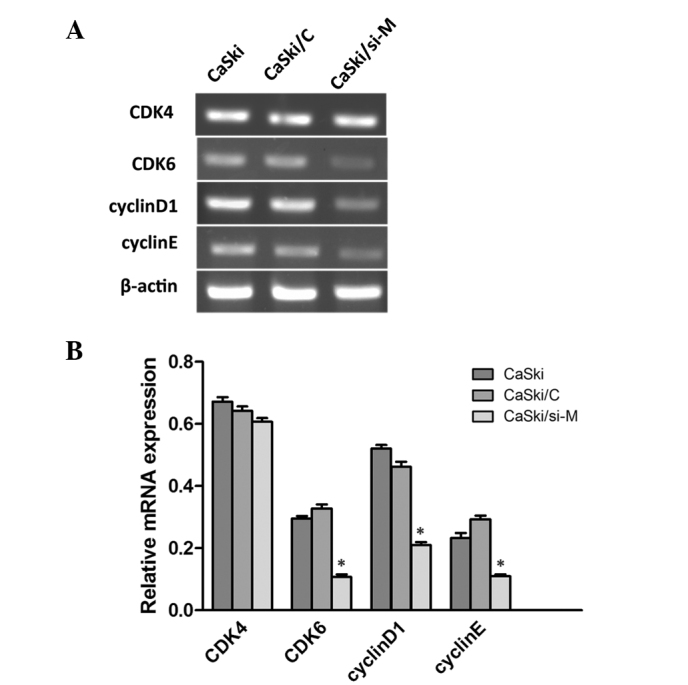
Downregulation of MALAT1 induced a decrease in the expression of cyclinD1, cyclinE and CDK6 in CaSki cells. (A) Images of semi-quantitative reverse transcription-polymerase chain reaction. (B) Bar graph of the relative expression of cyclinD1, cyclinE, CDK4 and CDK6 in CaSki, CaSki/C and CaSki/si-M cells. β-actin was used as a loading control. ^*^P<0.05, compared with CaSki. MALAT1, metastasis-associated lung adenocarcinoma transcript 1; CDK4/6; cyclin-dependent kinase 4/6.

To further examine the cause for the decreasing of cell viability, analysis of the cell cycle was conducted in CaSki cells. As revealed in Fig. 6, there was a significant decrease in S phase cells in CaSki/si-M cells compared with CaSki/C or CaSki/si-M, indicating that downregulation of MALAT1 inhibited the cell cycle at the G1/S transition. Following this, semi-quantitative RT-PCR was performed to investigate whether cell cycle associated molecules were affected by MALAT1. As revealed in Fig. 7, cyclinD1, cyclinE and cyclin-dependent kinase 6 (CDK6) were decreased in CaSki/si-M cells compared with the mock and control groups, while CDK4 was indistinguishable. These findings indicate that MLAT1 may increase cell proliferation by upregulating cyclinD1, cyclinE and CDK6.

### Knockdown of HPV16 E6/E7 reduces MALAT1 expression

**Figure 8:**
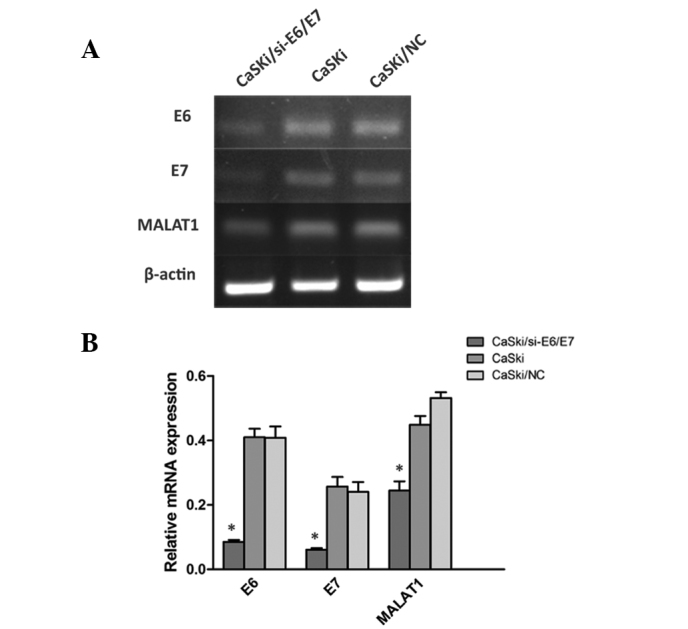
Knockdown of HPV16 E6/E7 reduced MALAT1 expression in CaSki cells. (A) E6, E7 and MALAT1 expression were measured by reverse transcription-polymerase chain reaction following transfection with E6/E7 interference sequence. (B) Bar graph of the relative expression of E6, E7 and MALAT1. β-actin was used as a loading control. ^*^P<0.05, compared with CaSki. MALAT1, metastasis-associated lung adenocarcinoma transcript 1.

To identify the possible factors inducing MALAT1 deregulation in cervical cancer, HPV E6/E7 was incorporated into the investigations due to its key role in cervical lesions. HPV16 E6/E7 shRNA vector GV102 targeting sequence GCAACAGTTACTGCGACGT (GeneChem, Shanghai, China) were transfected into HPV16-positive cell lines (CaSki cells). It was identified that the expression of MALAT1 was reduced with the E6/E7 downregulation in CaSki cells (Fig. 8). These results indicate that the HPV16 E6/E7 gene is involved in the upregulation of MALAT1 in cervical cancer.

### MALAT1 expression in clinical HPV-positive normal cervical squamous cells and lesions

**Figure 9:**
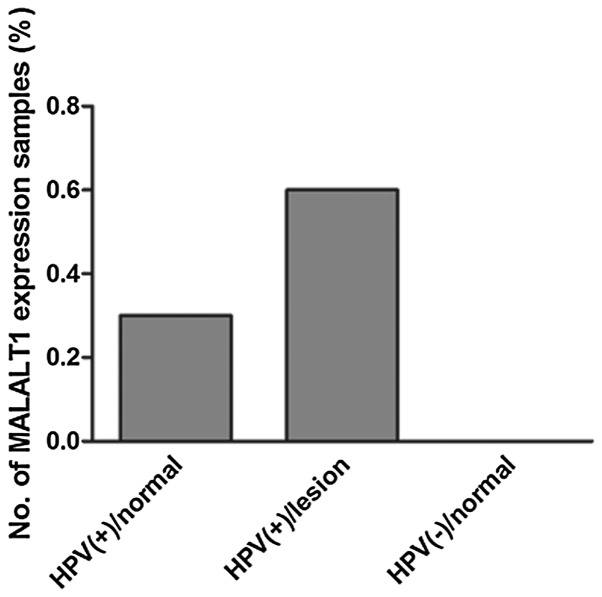
Number of samples expressing MALAT1 in clinical HPV-positive normal cervical squamous cells and lesions and HPV-negative normal cervical squamous cells. MALAT1, metastasis-associated lung adenocarcinoma transcript 1; HPV, human papilloma virus.

To further identify the correlation between MALAT1 and HPV, 64 cases of clinical cervical squamous cell samples were collected and MALAT1 expression was identified in 6/18 cases in HPV-positive cervical normal cells and 14/22 cases in HPV-positive cervical lesion specimens; while in all HPV-negative normal cervical squamous cells (n=24), MALAT1 expression was not detected (Fig. 9). This suggested that HPV is one of the important factors leading to MALAT1 activation in cervical SCC.

## Discussion

With the development of genomic microarrays and whole genome and transcriptome sequencing technologies, it has been revealed that ≥90% of the genome is actively transcribed, but <2%of the total genome encodes for functional proteins (27,28). These non-coding transcripts were previously argued to be spurious transcriptional noise, but have now been identified to have an increasingly important role in both normal development and disease, particularly in cancer initiation through interaction with protein-coding genes (29). MALAT1 is a lncRNA transcribed by RNA polymerase II (RNA pol II) and is not polyadenylated. It may be spliced to a 61-nt tRNA-like transcript and a longer transcript with a length of 6.7 kb. The 61-nt transcript may be exported to the cytoplasm the function of which remains unclear. The longer transcript was retained in nuclear speckles and were capable of regulating gene expresseion through interaction with SR (30). Several studies have reported that MALAT1 has a critical role in cancer development, including in lung, liver, breast and cervical cancer (16,21,23,31). The function of MALAT1 in cervical cancer was initially studied in our laboratory; however, the molecular mechanism in cell growth and the factors inducing MALAT1 upregulation were unclear. Furthermore, due to the established link between cervical cancer and HPV infection, elucidating the correlation among MALAT1, HPV and cervical cancer warranted further investigation.

In the present study, the expression of MALAT1 was detected in cervical cancer cell lines (HeLa, CaSki, SiHa and HCC94) and immortal human keratinocytes (HaCaT cells) using RT-PCR. HaCaT cells are a type of normal epithelial cell, so were utilized as a negative control. The results indicated that MALAT1 is expressed in cervical cancer but not in normal epithelial cells, and further study in normal cervical cell samples also demonstrated this. To study the functional role of MALAT1, stable MALAT1-underexpressing and -overexpressing cell lines were constructed in CaSki and HaCaT cells, respectively. A series of *in vitro* investigations indicated that the migration capability was evidently altered in CaSki/si-M, HaCaT/M1 and HaCaT/M2 compared with the control. The growth capability was decreased in CaSki/si-M while no alteration was observed in HaCaT/M1 and HaCaT/M2 compared with HaCaT. The explanation for this phenomenon may be due to the fact that the function fragments of MALAT1 in cell growth are different. A study in colorectal carcinoma demonstrated that a fragment spanning nucleotides 6918–8441 provides growth and proliferative advantage (25). To find the molecules associated with cell growth, the cell cycle of CaSki/si-M cells were analyzed and it was identified that the cells were arrested in G1 phase following MALAT1 downregulation. The cell cycle is controlled by numerous mechanisms ensuring correct cell division. The present study focused on the regulation of CDK by cyclins, and it was revealed that G1/S transition regulation molecules cyclinD1, cyclinE and CDK6 were decreased significantly in CaSki/si-M cells. These results suggest that MALAT1 may regulate cell proliferation through the P16INK4A/CDKs/RB pathway (32), while further studies are required to further elucidate this.

Since HR-HPV infection was an indispensable factor for cervical cancer and precancerous lesions, and numerous studies have demonstrated that HPV may target a group of molecules, including mTOR, miR-29 and MAGI-1 (33–35), it was hypothesized that HPV may be a key factor inducing MALAT1 expression in cervical SCC. MALAT1 expression was detected in 64 cases of HPV-positive or -negative cervical squamous cells, and it was identified that MALAT1 was expressed in ~30% of the HPV-positive normal cells and ~60% of the HPV-positive cervical lesion cells, but not in HPV-negative normal cells. Currently, the two main methods for cervical cancer screening are HPV testing and liquid-based cytology, both of which are associated with certain limitations (36–38). Joint inspection is a tendency based on the complete consideration of age, cost, positive rate and future efficiency. Our further study in HPV16-positive cells using shRNA targeting HPV16 E6/E7 proved that MALAT1 is a target of HPV. Therefore, MALAT1 may be a potential screening and therapeutic target for the treatment of cervical cancer. Further studies are required to analyze the molecular mechanisms of MALAT1 in cervical tumorigenesis, using more clinical samples to further analyze the correlation among MALAT1, HPV and cervical cancer progression.
